# Detection of erythroid progenitors and erythrocytopathies in patients with severe COVID-19 disease

**DOI:** 10.15537/smj.2022.43.8.20220311

**Published:** 2022-08

**Authors:** Mamdouh A. Allahyani, Abdulelah A. Aljuaid, Mazen M. Almehmadi, Ahmad A. Alghamdi, Ibrahim F. Halawani, Abdullah F. Aldairi, Ahmad M. Alharbi, Mohammad H. Albshri, Abdulqader A. Mutwalli, Ayman S. Alhazmi

**Affiliations:** *From the Department of Clinical Laboratory Sciences (Allahyani, Aljuaid, Almehmadi, Alghamdi, Halawani, Alharbi, Alhazmi), College of Applied Medical Sciences, Taif University, Taif; from Department of Laboratory Medicine (Aldairi), Faculty of Applied Medical Sciences, Umm Al-Qura University, Makkah; and from the Department of Molecular Biology (Albshri, Mutwalli), the Regional Laboratory, Ministry of Health, Makkah, Saudi Arabia.*

**Keywords:** erythroid progenitor cells, CD markers, SARS-CoV-2, COVID-19

## Abstract

**Objectives:**

To assess the effect of severe acute respiratory syndrome coronavirus-2 (SARS-CoV-2) infection on erythropoiesis and red blood cells (RBC) surface markers by evaluating erythroid progenitor cells (CD [cluster of differentiation]71^+^/CD235a^+^) and RBC surface markers (CD235a and CD36), together with various hematological parameters.

**Methods:**

This case-control study includes 47 participants recruited in the study: 30 patients with coronavirus disease 2019 (COVID-19) and 17 healthy individuals. The COVID-19 patients were recruited from the intensive care unit (ICU) of various hospitals in Makkah, Saudi Arabia. Blood samples were collected during July and September 2021. Red blood cells indices were measured using a CBC analyzer. The expression of CD235a, CD71, and CD36 was obtained using flow cytometry technique. The unpaired t-test was conducted to evaluate the differences in these markers in COVID-19 patients and healthy individuals.

**Results:**

The data showed that more than half of the COVID-19 patients were anemic (64%). Expansion of erythroid progenitors (CD71^+^/CD235a^+^) was detected in the COVID-19 patients. Analysis of the expression of RBC surface markers, such as CD235a and CD36, showed that SARS-CoV-2 was associated with significantly higher expression of these markers in COVID-19 patients.

**Conclusion:**

Severe acute respiratory syndrome coronavirus-2 promoted the expansion of erythroid progenitors in the peripheral blood of COVID-19 patients. In addition, the expression of RBC surface markers was higher in COVID-19 patients. The expansion of erythroid progenitors and alteration of RBC surface markers can contribute to erythrocytopathies observed in severe COVID-19 patients and can therefore be used as prognostic factors.

**T**he disease caused by a novel virus named severe acute respiratory syndrome coronavirus 2 (SARS-CoV-2), called coronavirus disease 2019 (COVID-19) was first reported in Wuhan, China in December 2019.^1,2^ In March 2020, the disease was announced as a pandemic disease globally.^3^ Pneumonia infections caused by COVID-19 have since been increasing and endanger both people’s health and the international economy.^4^ Some patients infected with SARS-CoV-2 require hospitalization or admission to the intensive care unit (ICU).^5^ However, the majority of patients are asymptomatic or exhibit mild symptoms.^5^ Coronavirus disease-19 is associated with many symptoms, including shortness of breath, fever, dry cough, and loss of taste or smell.^6^ In a subgroup of critically ill COVID-19 patients, hypoxia develops, leading to respiratory organ dysfunction, suggesting that red blood cells (RBCs) are affected, as RBCs are responsible for the oxygen delivery to all tissues.^7,8^ In normal circumstances, erythropoietin hormone is produced during hypoxia to stimulate erythroid progenitors in the bone marrow to produce more erythrocytes, which are then released into the circulation.^9^ However, hemoglobin is low and thrombocytopenia and anemia had been observed in severe cases of COVID-19 patients.^9,10^ Why anemia is developed in those patients is not fully understood, although these data suggested that SARS-CoV-2 can influence erythroid and megakaryocytic lineages in patients with COVID-19 disease.^9^ Anemia, coagulation, and thrombotic events in COVID-19 patients can contribute to high mortality.^11^

Although RBC counts and hemoglobin levels have been evaluated in COVID-19 patients, there is limited information on the direct effect of SARS-CoV-2 infection on erythropoiesis, erythroid progenitors, and RBC structural proteins such as CD235a [cluster of differentiation 235a], and CD36.^10^ Erythroid progenitors are defined as CD71^+^ and CD235a^+^.^7^ Cluster of differentiation 71 is a transferrin receptor expressed at all stages of erythroid development and absent in mature erythrocytes. However, CD235a (glycophorin A [GPA]) is expressed in all lineages and mature erythrocytes.^12^ Previous research reported that the expression of CD235a^+^ cells was higher in hypoxia compared with normoxia.^12,13^ It was indicated that the disruption of erythropoiesis may be an indirect effect of hyper inflammation observed in severe cases of COVID-19 disease.^14^ Glycophorin A (CD235a) is one of the most abundant proteins in RBC membranes. Glycophorin A (CD235a) is a sialoglycoprotein shown to reduce RBC aggregation in the circulation.^15^ Research has also shown that erythrocytes lacking GPA and glycophorin B are not susceptible to *Plasmodium falciparum* (*P. falciparum*) parasite infection, suggesting that GPA may be a receptor for *P. falciparum*.^16^

A few studies have observed the effect of SARS-CoV-2 on RBC’s structural proteins.^6,7,17^ Recent proteomics analyses detected angiotensin and angiotensin-converting enzyme receptor 2 (ACE2)-interacting proteins on the surface of RBCs in patients with COVID-19 disease.^6^ Although RBCs cannot support viral replication, this finding suggests that the virus may target RBCs.^6^ It was shown that SARS-CoV-2 infects RBCs via an interaction between the Band 3 protein of RBCs and a spike protein in the SARS-CoV-2 virus.^17^ Damage to structural proteins and changes in RBC membrane lipids have been reported in COVID-19 patients.^7^ In a previous study, the sizes of erythrocytes were increased in COVID-19 patients.^11^ In another study, the RBC’s distribution width (RDW) was elevated and associated with a higher mortality rate.^18^ In addition, a study reported that SARS-CoV-2 infection caused damage to the erythrocyte proteome.^11^ Furthermore, heme metabolism was inhibited following the SARS-CoV-2 infection.^7^ An increase in the levels of structural RBC proteins, including the cytosolic N-terminus of band 3 (AE1), spectrin alpha (SPTA1), and ankyrin (ANK1) has also been found in COVID-19 patients.^6^ These data indicate that SARS-CoV-2 affects various structural proteins, involved in the integrity of RBC membrane. However, only a few studies have shown the effect of the virus on CD235a and CD36.^12^

Cluster of differentiation 36 is expressed in all erythroid progenitors and mature RBCs, but CD36 has never been evaluated in COVID-19 patients.^19,20^ Cluster of differentiation 36 is a glycosylated protein that binds to thrombospondin, Von Willebrand factor, and fibronectin.^20^ It is an adhesion molecule for platelets, monocytes, and endothelial cells. CD36 acts as a receptor during infection with *P. falciparum*.^20^ Cluster of differentiation 36 is highly expressed in sickle cell disease, and it plays an essential role in the adhesion of sickle cells to endothelial cells.^21^

The RBC biology, expansion of erythroid progenitors, and structural protein alterations may be associated with erythrocytopathies observed in critically ill COVID-19 patients. This study aimed to evaluate the effects of SARS-CoV-2 infection on erythropoiesis, erythroid progenitors, and RBC’s surface markers, including CD71^+^/CD235a^+^, CD235a, and CD36, in addition to other different hematological parameters.

## Methods

In this case-control study, 47 participants were included. A total of 30 COVID-19 patients and 17 healthy individuals. The age range was 49-65 years old. Clinical data were obtained from ICU patients of various hospitals in Makkah, Saudi Arabia. The inclusion criteria include all the patients who had been diagnosed with COVID-19 using the real-time quantitative reverse transcription-polymerase chain reaction assay based on a nasopharyngeal or throat swab. Only patients admitted to the ICU were included in the study. Any patient diagnosed with anemia prior to the study has been excluded from the study. Many patients required mechanical ventilation. All the patients and healthy controls recruited in the study have signed informed consent. The study was performed according to the principles of the Helsinki declaration. The study was approved by the Institutional Review Board of the directorate of health affairs in Makkah City (registration number: HAP-02-T-067; approval number 427). Blood samples were collected between July and September 2021. A complete blood count (CBC) was performed on all samples using an automated CBC analyzer (Mindray BC-2800 analyzer, Shenzhen, China). Only RBC indices were included in this study. The RBC indices were measured and evaluated according to World Health Organization (WHO) guidelines.

### Red blood cell isolation and flow cytometry

Peripheral blood from the hospitalized COVID-19 patients and healthy controls was collected in ethylenediaminetetraacetic acid tubes and processed within 24 hours. The blood samples were layered carefully with one volume of Ficoll-Paque (GE Healthcare, Little, Chalfont, Buckinghamshire, UK), centrifuged at 2000 rpm for 20 minutes with the brake off at 4°C. Peripheral blood mononuclear cells were isolated from whole blood by density centrifugation using the Ficoll-Paque gradient and kept for other studies. The pellet supernatant (RBCs) was stained with different surface markers. Firstly, 1 μL of RBCs was washed with phosphate buffer saline (PBS) and stained with anti CD235a (phycoerythrin), CD36 (allophycocyanin) and CD71 (fluorescein isothiocyanate) for 30 minutes in the dark at a 1:100 ratio. The pellet cells were then washed with PBS and resuspended in 200μl of PBS. The samples were analyzed using a BD FACSCanto II system (BD Bioscience, San Jose, CA, USA) and FACSDiva software, version 6 (BD Biosciences). The RBCs were gated based on side scatter and forward scatter. The data were analyzed using FlowJo software version 7.10 (Tree Star, Ashland, Oregon, USA).

### Statistical analysis

GraphPad Prism software, version 6.04, was used to analyze the data (La Jolla, CA, USA). Patients’ data were presented as mean and standard deviation (SD). According to the data distribution, differences in the RBC surface markers between the COVID-19 patients and healthy controls were analyzed by an unpaired t-test, where the level of significance was set at *p*<0.05.

## Results

The patient’s mean age enrolled in the study was 57±6.1 years as patient data were summarized (Table 1). The hematological parameters revealed several RBC abnormalities. More than half of COVID-19 patients (64%) were anemic. Low RBC counts, low hemoglobin, and low hematocrit levels were observed in the COVID-19 patients. The red blood cell distribution width (RDW) was slightly elevated in the COVID-19 patients, pointing to RBC shape changes. However, the mean corpuscular volume (MCV), mean corpuscular hemoglobin (MCH) and mean corpuscular hemoglobin concentration (MCHC) levels were normal.

**Table 1:** - Patient sample characteristics.

Parameter	Healthy (mean ± SD)	Patients (mean ± SD)
Age (years)	46 ± 3.7	57 ± 6.1
***Gender (n,%)***		
Male	11 (64.7)	18 (60.0)
Female	6 (35.3)	12 (40.0)
RBCs (mean ± SD)x10^6^ /ml	4.9 ± 0.4	3.8 ± 1.4
Hemoglobin (g/dL)	14.2 ± 1.1	10.8 ± 2.0
Hematocrit	44.2 ± 3.5	33.5 ± 3.6
MCV (fL)	87.6 ± 2.5	85.2 ± 3.7
MCH (pg)	29.8 ± 1.7	27.8 ± 2.6
MCHC (g/dL)	34.1 ± 1.3	32.9 ± 2.4
RDW	12.2 ± 1.1	14.7 ± 1.8

RBCs: red blood cells, MCV: mean corpuscular volume, MCH: mean corpuscular hemoglobin, MCHC: mean corpuscular hemoglobin concentration, RDW: red blood cell distribution width, SD: standard deviation

**Figure 1:**
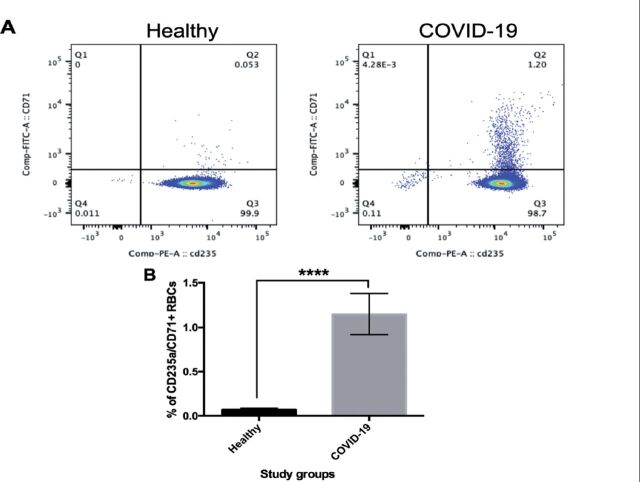
- Detection of erythroid progenitors (CD235a^+^/CD71^+^) in patients with coronavirus 2019 (COVID-19) compared with healthy individuals. Red blood cells (RBCs) were isolated from peripheral blood of the COVID-19 patients and healthy individuals. The cells were then stained with anti-CD71 (FITC) and CD235a (PE) and visualized using flow cytometry. A) Dot plots represent the percentage of CD71 and CD235a in the samples obtained from the healthy individuals and COVID-19 patients. B) The percentage of CD235a/CD71-positive cells is presented. Data were analyzed by an unpaired t-test. Values represent mean±SD. ^****^ *p*=0.0001.

To investigate the effect of SARS-CoV-2 on erythroid progenitor cells in the peripheral blood of the COVID-19 patients, flow cytometry was performed. Our results showed that erythroid progenitor cells (CD71^+^/CD235a^+^) were seen in the COVID-19 patients, with negligible expression in the control group (Figure 1A). The expression of CD71^+^/CD235a^+^ was significantly higher in the COVID-19 patients than in the control group (*p*≤0.0001, Figure 1B).

**Figure 2:**
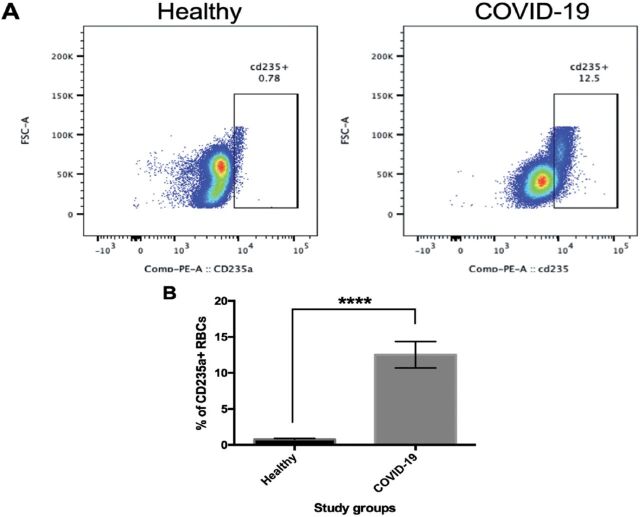
- Expression of cluster of differentiation (CD)235a in patients with coronavirus 2019 (COVID-19) compared with healthy individuals. Red blood cells (RBCs) were isolated from peripheral blood of the COVID-19 patients and healthy individuals. The cells were then stained with anti-CD235a (PE) and visualized using flow cytometry. A) Dot plots represent the percentage of CD235a in the samples obtained from the healthy individuals and COVID-19 patients. B) The percentage of CD235a-positive cells is presented. Data were analyzed by an unpaired t-test. Values represent mean ± SD. ^****^ *p*=0.0001.

Having shown that the RDW was elevated in the COVID-19 group, the expression of some RBC surface markers, such as CD235a, and CD36 was examined using flow cytometry. As CD235a is normally expressed on mature RBCs, we investigated whether this surface marker was altered in COVID-19 patients. The results revealed a higher rate of CD235a positivity in the COVID-19 group as compared with that in the control group (Figure 2A), and this was significantly different (*p*≤0.0001, Figure 2B).

**Figure 3:**
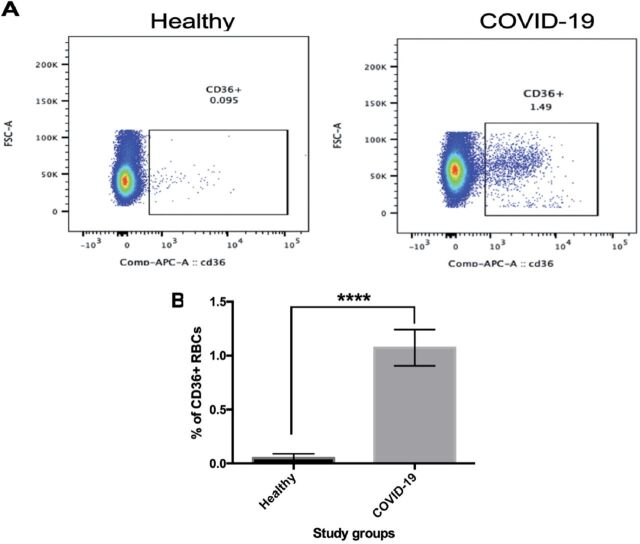
- Comparison of cluster of differentiation (CD) 36 expression in patients with coronavirus 2019 (COVID-19) and healthy individuals. Red blood cells (RBCs) were isolated from peripheral blood of the COVID-19 patients and healthy individuals. The cells were then stained with anti-CD36 (APC) and visualized using flow cytometry. A) Dot plots represent the percentage of CD36 in the samples obtained from the healthy individuals and COVID-19 patients. B) The percentage of CD36-positive cells. Data were analyzed by an unpaired t-test. Values represent mean±SD. ^****^ *p*=0.0001.

Finally, the analysis of CD36 expression results showed increased expression of CD36 in the COVID-19 patients (Figure 3A). CD36 expression was significantly different in COVID-19 than in healthy controls (*p*≤0.0001, Figure 3B).

## Discussion

This study was carried out to investigate the impact of SARS-CoV-2 infection on the expansion of erythroid progenitor cells (CD71^+^/CD235a^+^), and RBC surface markers (CD235a^+^ and CD36^+^). In addition, this study aimed to examine whether various hematological parameters are frequently altered in severe cases of COVID-19 patients. Abnormalities in RBC indices and alterations in RBC surface markers can be used to predict the severity of disease progression in COVID-19 patients.

Our data shows that 64% of the COVID-19 patients in the study were anemic. These results are consistent with those of other studies, which reported that anemia was common among COVID-19 patients, especially hospitalized patients, and associated with high mortality.^10,22^ Anemia in COVID-19 patients has also been reported to be associated with worse outcomes.^23^ However, the COVID-19 patients in our cohort were normocytic and normochromic as indicated by the normal levels of MCV, MCH, and MCHC. These findings also agree with those of another study.^24^

A previous study showed that a higher RDW was associated with a higher mortality rate in COVID-19 patients, as our data showed the RDW was slightly elevated that are in accordance with this finding.^18^ These data pointed to variations in RBC shapes in patients infected with COVID-19 disease. The increase in the RDW in patients with COVID-19 suggested that other morphological abnormalities might be seen in those patients. Previous research reported a few nucleated RBCs and cytoplasmic vacuolation of monocytes in the circulating blood of COVID-19 patients.^24^ However, another study failed to detect nucleated RBCs, suggesting that their presence may be related to the severity of the disease.^25^ Other abnormalities, such as pyknotic cells, broken cells, pseudo Pelger-Huët cells, abnormal lymphocytes, abnormal monocytes, leukoerythroblastic reactions, and smudge cells, have also been detected in patients with COVID-19 disease.^25,26^ Furthermore, schistocytes (red blood cell fragments) have been detected in the peripheral blood of some COVID-19 patients.^27^ Previous research indicated that the decline in hemoglobin in COVID-19 patients was associated with the appearance of nucleated RBCs in the peripheral blood.^14^ The presence of circulating nucleated red cells in the peripheral blood suggests that SARS-CoV-2 infection induces aberrant erythropoiesis.

The appearance of nucleated RBCs in COVID-19 patients prompted us to examine whether erythroid progenitor cells (CD71^+^/CD235a^+^) were present in the circulating blood of COVID-19 patients. Our data showed that the erythroid progenitor cells (CD71^+^/CD235a^+^) were significantly higher in the COVID-19 group than in the control group. Recent studies reported similar findings.^7, 14^ Specifically, they have shown that the expression of CD71^+^/CD235a^+^ was elevated in severe COVID-19 cases more than in mild cases.^7,14^ Another study has observed an elevation of erythroid progenitor cells and megakaryocytes in severe COVID-19 cases.^13^ Typically, these cells reside in the bone marrow. Thus, their presence in the peripheral blood of COVID-19 patients requires further investigation. However, various explanations have been put forward to explain the increase in erythroid progenitors in the circulation in COVID-19 patients. The finding of angiotensin and ACE2-interacting proteins on RBCs’ surface suggests that the virus affects RBCs.^6^ Furthermore, it was shown that SARS-CoV-2 infects RBCs via an interaction between the band 3 protein of RBCs and a spike protein in the SARS-CoV-2 virus.^17^ In addition, it was reported that heme metabolism was inhibited following SARS-CoV-2 infection.^7^ They also found a colocalization between ACE2 and erythroid progenitors.^7^ Another study showed that high expression of ACE2 was observed during erythropoiesis.^14^ They also found that only erythroid progenitors express ACE2 but normal hematopoietic stem cells do not. In addition, they detected several SARS-CoV-2 genes in erythroid progenitors following the infection, including nucleocapsid and envelope genes.^14^ These data indicated that SARS-CoV-2 may invade RBCs, resulting in stress erythropoiesis to compensate for the low oxygen levels observed in those patients leading to the abundance of erythroid progenitors in the circulation.

Higher levels of CD235a were observed in severe cases of COVID-19 patients in our study. This finding agreed with that of another study, which showed that CD235a^+^/CD71^-^ cells were higher in COVID-19 patients than those in healthy controls.^7^ Some studies investigated the effect of SARS-CoV-2 on RBC’s structural proteins.^6,7,17^ One study has reported alterations in structural RBC surface markers.^6^ For example, researchers detected an increase in the levels of different structural RBC proteins, including the cytosolic N- terminus of band 3 (AE1), spectrin alpha (SPTA1), and ankyrin (ANK1) in COVID-19 patients.^6^ However, fewer studies were focused on the effect of SARS-CoV-2 on CD235a.^7^

Finally, there are limited data on the expression of CD36 in COVID-19 patients. Previous research showed that CD36 acts as a receptor during other infections, such as those associated with *P. falciparum*.^20^ In addition, CD36 is known to play a crucial role in the adhesion of sickle cells to endothelial cells.^21^ In the current study, CD36 expression in severe COVID-19 cases was higher than in healthy controls. More research is required to clarify the exact role of CD36 during COVID-19 infection.

### Study limitations

There is a small number of patients enrolled in the study. In addition, only one group of COVID-19 patients (severe cases) were included in the study and compared with healthy individuals. Including more study groups (moderate and mild cases of COVID-19 patients) would be valuable. Also, the levels of erythroid progenitors should be monitored over time for patients during their study at the hospital.

In conclusion, we evaluated the effect of SARS-CoV-2 infection on RBC indices, erythroid progenitors (CD71^+^/CD235a^+^), and RBC surface markers (CD235a and CD36). Our data suggested that more than half of the COVID-19 were anemic. In our study, we found many RBC abnormalities in the circulating blood of the COVID-19 patients. These included increased expression of RBC structural proteins (CD235a and CD36), and expansion of erythroid progenitors. These findings can be associated with diminished production of erythrocytes, leading to the development of anemia. Together with hypoxia and anemia observed in those patients, these manifestations may be linked with the disease severity and mortality and might have potential as prognostic factors in the management of severely ill COVID-19 patients.
